# Bacterial Genomes: Habitat Specificity and Uncharted Organisms

**DOI:** 10.1007/s00248-012-0017-y

**Published:** 2012-03-07

**Authors:** Francisco Dini-Andreote, Fernando Dini Andreote, Welington Luiz Araújo, Jack T. Trevors, Jan Dirk van Elsas

**Affiliations:** 1 Department of Genetics, “Luiz de Queiroz” College of Agriculture, University of São Paulo, Piracicaba, Brazil; 2 Department of Soil Science, “Luiz de Queiroz” College of Agriculture, University of São Paulo, Piracicaba, Brazil; 3 Institute for Biomedical Sciences, University of São Paulo, São Paulo, Brazil; 4 School of Environmental Sciences, University of Guelph, Guelph, ON Canada N1G 2W1; 5 Department of Microbial Ecology, Centre for Ecological and Evolutionary Studies, University of Groningen, Nijenborgh 7, 9747 AG Groningen, The Netherlands

## Abstract

The capability and speed in generating genomic data have increased profoundly since the release of the draft human genome in 2000. Additionally, sequencing costs have continued to plummet as the next generation of highly efficient sequencing technologies (next-generation sequencing) became available and commercial facilities promote market competition. However, new challenges have emerged as researchers attempt to efficiently process the massive amounts of sequence data being generated. First, the described genome sequences are unequally distributed among the branches of bacterial life and, second, bacterial pan-genomes are often not considered when setting aims for sequencing projects. Here, we propose that scientists should be concerned with attaining an improved equal representation of most of the bacterial tree of life organisms, at the genomic level. Moreover, they should take into account the natural variation that is often observed within bacterial species and the role of the often changing surrounding environment and natural selection pressures, which is central to bacterial speciation and genome evolution. Not only will such efforts contribute to our overall understanding of the microbial diversity extant in ecosystems as well as the structuring of the extant genomes, but they will also facilitate the development of better methods for (meta)genome annotation.

## Introduction

Next-generation sequencing (NGS) [22] is currently revolutionizing our capabilities to deepen the understanding of the ecology and diversity of microorganisms in natural settings. However, there is a current consensus that the technology is progressing so fast that a much required basis or background for the sound interpretation of the massive sequence information is lacking. Key to this is the generally perceived lack of sufficient information on the genomes of representative taxa in each of the over 50 phyla that currently make up the bacterial tree of life [14, 16]. Moreover, in cases where genome sequence information is available, there is often a lack of broad information on the genomic variation within that species. Hence, a prime objective of current ecologically oriented sequencing projects should be to assign whole-genome sequences to each of the branches of the current tree of bacterial radiation, as well as broaden our knowledge of the within-species variation at each of the species branches of the tree. There is also a need for a proper correlation of the genomes of particular bacteria to the environment from where they were obtained (e.g. both geographically defined locations and the same and different environmental locations; rhizosphere, nonrhizosphere, contaminated waste sites, pathogens in patients, water, food and hospitals) and to assess if there is a “habitat- or niche-specific genome” which is recognizable from particular environmentally relevant features or signatures (Box 1).

The first benefit that may come from a more robust representation of the tree of life at the genome sequence level would be an improvement of the analytical power provided by both genomics and metagenomics projects. Thus, the occurrence of patterns (and eventually standards) in genome organisation across distinct microbial species and habitat-specific drivers of genome evolution will be better understood and may assist in transformative research discoveries. The key phenomenon of within-species variation implies that for highly variable genomes, which result in large and often still open pan-genomes, as many well-selected strain genomes as possible should be sequenced and annotated. Thus, a survey of *Escherichia coli* genomes recently revealed huge within-species variation, leading to the concept of a physiologically and ecologically very adaptable species [13, 21]. Although there is a paucity of current strong evidence for this, it is possible that the intrinsic patterns of organisation of such genomes have evolved differently for particular habitats or niches. Moreover, when a single strain is followed through time (i.e. tens of thousands of generations in a laboratory population), considerable variation can be observed at the genome level [3]. From these and other studies, it has become apparent that bacterial genomes are inherently dynamic with some constancy and yet capable of changes during evolution. However, at the same time, they must adhere to generic rules of efficiency (of replication, repair and transcription)—determining fitness—that govern their makeup.

Previous and current initiatives to enhance the number of sequenced bacterial genomes in databases have based their selection mainly on the relevance of the bacterium from a public health or applied industrial perspective. Also, evolutionary relationships that were perceived to be most relevant have been guiding the selection of organisms [21, 27]. Moreover, genome sequencing projects have mostly been based on cultured representatives of the extant microbial diversity in most ecosystems, which is actually known to quite poorly represent the true diversity of species that abound in the environment. Thus, in the light of the current quest for better microbial cultivation methodologies, targeting as-yet uncultured microbes for genome sequencing is urgent, as it will:

1. fill gaps in the information on the sequences of genomes of organisms from poorly covered clades,
2. (upon sequencing of multiple genomes per species) allow an understanding of the within-species diversity per newly covered species, and
3. lead to a solid basis for the development of better strategies for microbial cultivation.
4. decrease knowledge fragmentation and contribute to knowledge fusion approaches

## Box 1. Habitat-Specific Genomes—Fact or Fiction?

**Figure 1:**
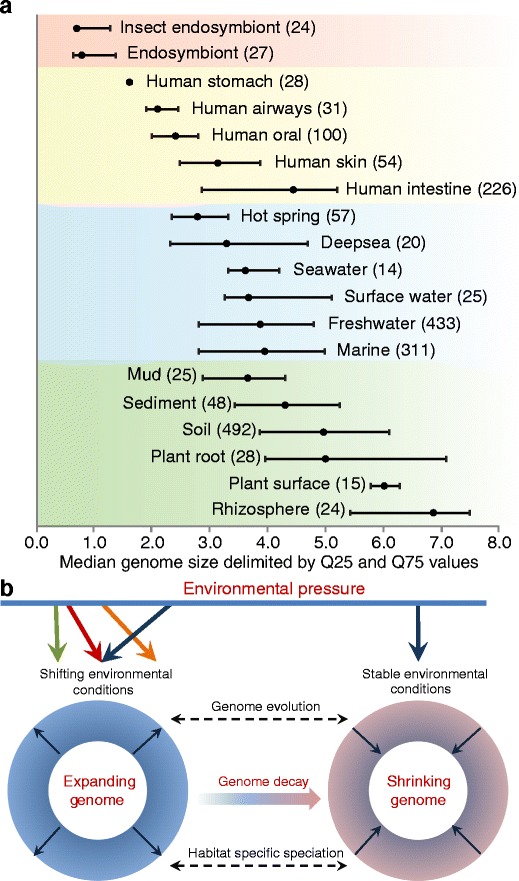
**a** Range of bacterial genome sizes in various habitats delimited by quartile 25 and 75 values. *Numbers in parentheses* indicate the amount of analysed genomes. Data were extracted from the Genomes OnLine Database (GOLD) [4] in September 2011. **b** Proposed model for microbial genome evolution and habitat-specific speciation

Recent advances in meta(genome) data processing have provided knowledge on factors that modulate microbial speciation and genome evolution. The link is remarkable between the functional complexity of microbial genomes and the habitats where organisms survive and reproduce. Recently, Raes et al. [17] described an effective model to determine the effective genome size in metagenomics data. Intrinsic to this model is the concept that each habitat harbours a specific range of genome sizes which stand in relation to the prevailing factors in the habitat (Fig. 1a). The concept has been used as a metric parameter to infer community diversity and complexity [2], in which longer average genome lengths correlate with a more complex and dynamic habitats. The hypothesis is that bacteria with larger genomes can easier cope with such conditions as they encode a larger metabolic and stress tolerance potential [18]. In fact, the evolution of microbial species is affected by the environmental pressures acting over time (Fig. 1b). A clear example of this is the massive genome reduction in bacteria that adapts to a mutualistic/symbiotic lifestyle [cf. 20], resulting in tiny, gene-dense genomes [5]. It remains to be seen whether habitat-specific patterns can be distinguished among different genomes within the species. Certainly, the concept of habitat-specific genomes highlights the role of surrounding environment acting at the core of genome speciation and the evolution of microbial species. The collection of contextual (meta)data, encompassing physical–chemical parameters and allocating the source of a sequence in terms of space and time, surely will allow a better interpretation of unknown genes and species, as well as gaining new insights into the known fraction [28].

## Metagenomics—The Need to Include Genome Sequences of Bacteria Representative for Natural Ecological Settings in Databases

**Figure 2:**
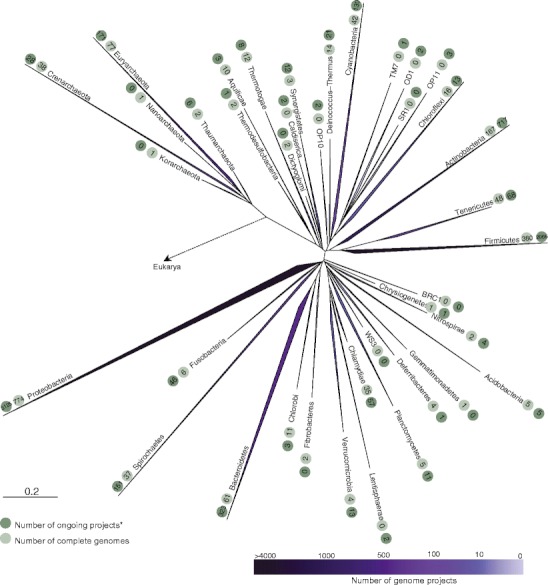
Phylogenetic distribution of microbial genome projects at the phylum level. Data were extracted from the Genomes OnLine Database (GOLD) [4] in September 2011. The phylogenetic distribution was constructed using Silva Ref SSU database release 104 (http://www.arb-silva.de). (*) encompass ‘incomplete’, ‘permanent draft’ and ‘target’ status at GOLD

**Figure 3:**
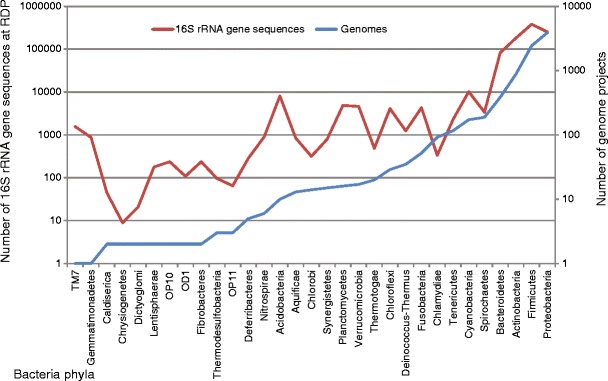
Number of 16S rRNA sequences at RDP (http://rdp.cme.msu.edu/)—representing environmental sequences—and total genome projects at GOLD—representing genomes, according to their phylogenetic distribution

A key issue of great current relevance is the metagenomics approach to ecosystem analyses [6, 23]. This approach has been expanding since 1999, mostly as a result of the power of NGS. While the generation of massive numbers of sequences from extant microbial communities appears promising to achieve a complete overview of the genetic profile in distinct environments [23], the analysis of these sequences and the proper assignment of DNA tags to their original owners in nature has emerged as a major challenge for bioinformatics (called the “computational bubble”). Our ability to properly correlate environmental genomic data to currently charted bacteria is strongly hindered by the lack of whole-genome sequences for many of the microorganisms dispersed along the phylogenetic tree of life (Fig. 2). Here, we posit that a major cause of this problem is that the basis of the current data set is in the subset of culturable Bacteria and Archaea. This, as stated by Gilbert et al. [7], is the underlying cause of our current inability to robustly annotate the major part of the genes found in environmental metagenomics data. Only up to 4% of the sequences were thus found to be identifiable to species [7]. The pool of hitherto-cultured microorganisms indeed vastly underrepresents the true scope of the microbial diversity found in most natural ecosystems. And, on top of this, we lack information on the within-species diversity (defining the pan-genome) across both the poorly accessed as well as most of the well-known organisms. This lack of representativeness can, for instance, be observed by comparing the number of 16S ribosomal RNA gene tags from each bacterial and archaeal phylum in the Ribosomal Database Project (RDP) database (mostly obtained from environmental samples) to the number of complete and ongoing genome sequencing projects *per phylum* (Fig. 3). To date, no large-scope sequencing project has been filed that aims to comprehensively cover the genomes of as-yet unculturable uncharted microorganisms. Not to speak of the members of the still underexplored rare biosphere, which might fall into the previous class, but might also have been missed by their sheer rarity [15, 19].

These issues of underrepresentation pose significant challenges to initiatives such as TerraGenome, which aims to sequence and characterize the soil microbial communities in one standard soil, Rothamsted Park grass, and then use the data set for worldwide comparative work [24], Earth Microbiome and Human Microbiome, which aims to describe the microbial communities that inhabit respectively Earth and the human body in a collaborative global effort. In such projects, large portions of the sequences generated remain unclassified as a result of the poor representation of particular, environmentally relevant, microbial taxa in genome databases, leaving them unidentified. An example of such underrepresented taxa is formed by the *Acidobacteria* in soil [10] and by the clade OP11 in marine systems [8]. The *Acidobacteria* are very diverse, currently encompassing around 30 species or candidate species. A lot is to be gained by including single genome sequences of each of the “species” into the database. Moreover, we do not understand the within-species variation across all of these acidobacterial taxa. Thus, addition of more sequence information on the basis of whole genomes is a real must to foster the developments in metagenomics of whole natural systems. Hence, it is proposed that the generation of a phylogenetically complete database of microbial genomes, including both culturable and unculturable microbial groups, will substantially contribute to the accurate affiliation of metagenomic sequences, representing a giant step forward in environmental microbiology. And, on top of this, there is a need to sequence not just one, but several to many members of each microbial species to cover the extant within-species genomic diversity, leading to the complete pan-genome. In this respect, bacteria are known to have either a tightly regulated genome or a highly variable one, resulting in either restricted (closed) or open pan-genomes [1, 11]. Also, there is a need to improve our skills in bioinformatics, yielding highly efficient bioinformatics tools that will allow processing of the terabase-sized data sets in a logical and workable manner.

## Available Tools

Our capacity to generate DNA sequences from virtually any environmental sample has achieved a level of efficiency where it is now virtually possible to sequence DNA up to the size of an entire human genome in a single day. However, the limitation of having to deal with unculturable and rare microbes is still present, hampering the preparation of the genetic material to be processed and sequenced. A possible solution lies in the use of intelligently selected cultivation approaches, in which often highly purified (but not 100% pure) cultures are possible targets for sequencing. Another solution is offered by technologies like Single Cell Genomics [9, 26], although this approach obviously also limits the scope of what can be achieved. In addition, singling out specific microbial groups from complex environmental samples is already possible by established technologies, including laser microdissection, flow cytometric assisted cell sorting, or Raman microspectroscopy [25]. Such separation could be achieved according to the phylogenetic affiliation of cells or by applying substrate use as a criterion, using stable isotopes [25]. Considering the available approaches, we propose that future microbial genomic sequencing projects be focused on those microbial groups that are poorly represented by the completed and ongoing genome surveys [cf 4]. The results from these new projects would contribute to a better description and understanding of the genome organisation across and within currently underrepresented bacterial species. In particular, the scope and impact of horizontal gene transfer on bacterial evolution need a much better focus, which can be gleaned from genome cross-comparisons [12]. A recent example revealed critical genomic regions that varied in accordance with function across several *Dehalococcoides* genomes [12]. Ideally, such cross-genome comparisons would allow the determination of the sizes of the pan-genomes across as many species per family and phylum as possible [1]. Finally, this type of analysis would facilitate a much improved interpretation of data from metagenomics projects through the specificity and increased understanding of the overall function of bacteria in natural milieus.

## Concluding Remarks

The purpose of this opinion is to promote developments that will spur the generation of genome sequence data that are beneficial to the interpretation of the currently available and future metagenomics data sets [6, 23]. One perspective is obviously that the existing barriers to data interpretation are overcome, another one that the description and analysis of patterns across bacterial genomes are facilitated [12]. Moreover, the detection of as-yet-to-be-described genes in distinct environmental bacteria would be facilitated. Overall, we argue in favour of the establishment of a consolidated platform for microbial genome and metagenome comparisons and metagenomics data affiliation. Such a platform would be web-based and would allow researchers to jointly set directions for the completion of the genome-based bacterial tree of life, allowing equal representation of all currently recognized branches. This ambitious project will certainly bring the same quantum leap forward in understanding the microbial diversity on the planet as the previous leap which was based on the use of direct molecular assessments on the basis of the 16S ribosomal RNA sequence as a marker suitable for charting the extant microbial diversity in ecosystems. In conclusion, we propose, as an important target for upcoming projects, the sequencing and annotation of the genomes of multiple members of the as-yet-uncultured and/or as-yet-uncharted natural microbiota, including that of members of the rare biosphere. The results from such an undertaking would lead to a wider description of genome organisation in environmental bacteria and to a more accurate annotation of current metagenomics data sets, constituting another (decadal) milestone in the field of environmental microbiology and forthcoming transformative discoveries.
